# Association between Probiotics and Modulation of Gut Microbial Community Composition in Colorectal Cancer Animal Models: A Systematic Review (2010–2021)

**DOI:** 10.1155/2023/3571184

**Published:** 2023-09-09

**Authors:** Shabnam Zeighamy Alamdary, Shahnaz Halimi, Akram Rezaei, Roghayeh Afifirad

**Affiliations:** Department of Microbiology, School of Medicine, Tehran University of Medical Sciences, Tehran, Iran

## Abstract

**Background:**

Colorectal cancer (CRC) is one of the most prevalent gastrointestinal malignancies and is considered the third major cause of mortality globally. Probiotics have been shown to protect against the CRC cascade in numerous studies.

**Aims:**

The goal of this systematic review was to gather the preclinical studies that examined the impact of probiotics on the alteration of gut microbiota profiles (bacterial communities) and their link to colorectal carcinogenesis as well as the potential processes involved.

**Methods:**

The search was performed using Scopus, Web of Science, and PubMed databases. Five parameters were used to develop search filters: “probiotics,” “prebiotics,” “synbiotics,” “colorectal cancer,” and “animal model.”

**Results:**

Of the 399 full texts that were screened, 33 original articles met the inclusion criteria. According to the current findings, probiotics/synbiotics could significantly attenuate aberrant crypt foci (ACF) formation, restore beneficial bacteria in the microbiota population, increase short-chain fatty acids (SCFAs), and change inflammatory marker expression.

**Conclusions:**

The present systematic review results indicate that probiotics could modulate the gut microbial composition and immune regulation to combat/inhibit CRC in preclinical models. However, where the evidence is more limited, it is critical to transfer preclinical research into clinical data.

## 1. Introduction

Colorectal cancer, a multifactorial gastrointestinal malignancy, is one of the most critical public health issues and the third leading cause of cancer mortality worldwide [1]. In recent years, the global prevalence of CRC has increased worryingly. In 2020, there were expected to be 1.93 million new CRC cases diagnosed and 0.94 million CRC-related deaths worldwide, accounting for 10% of global cancer prevalence (total 19.29 million new cases) and 9.4% of all cancer-related deaths (total 9.96 million deaths) [2]. Today, over 5.25 million (5-year prevalence) people are living with CRC globally. According to GLOBOCAN 2020 [3] estimates, there will be 1.15 million new cases of colon cancer and 0.7 million new cases of rectal cancer in 2020 worldwide. With continued growth, these numbers are expected to rise to 1.92 million and 1.16 million, respectively, in 2040. The gut microflora with bacteria as its predominant inhabitants helps the human immune system mature and maintain the natural barrier's integrity. In a healthy individual, the structure and immune function of the colorectal epithelium preserve a mutually beneficial relationship between the microbiota and the host [4]. Indeed, a healthy microbiota prevents the proliferation and colonization of pathogenic bacteria by covering intestinal niches and fighting for nutrients. Several factors, including gene mutations, family history, dietary compounds, and microbial dysbiosis, might contribute to the improvement of CRC disease [5]. Among them, increasing research shows that CRC development is strongly correlated with gut microbiota dysbiosis. Microbial dysbiosis is linked to the production of carcinogenic agents, as well as inducing inflammatory responses, secondary bile acid synthesis, and metabolic signals that lead to malignant alterations in epithelial cells and, eventually, the prevalence of CRC [6, 7]. CRC patients have considerably reduced intestinal microbiota diversity and clearly altered microbial abundance compared to healthy people [8]. These days, the advancement of next-generation sequencing technologies has made it easier to analyze microbial composition and diversity. In a healthy and normal gut, the most prevalent bacteria mainly are two phyla, *Firmicutes* and *Bacteroidetes,* which account for approximately 90% of the microbial system [9]. Currently, specific species such as *Fusobacterium nucleatum*, *Escherichia coli*, *Bacteroides fragilis*, *Peptostreptococcus stomatis, Parvimonas micra, and Campylobacter jejuni* are enriched in CRC [10], in contrast to some beneficial species such as *Bifidobacterium breve, Lactobacillus rhamnosus,* and *Akkermansia muciniphila* which are poor in CRC subjects. Furthermore, the metagenome sequencing and metabolomics combination demonstrated that the CRC-associated microbiome can be a source of harmful metabolites (e.g., L-2 hydroxyglutarate, succinate, and fumarate). For example, *B. fragilis*, *F. nucleatum*, and some Prevotellaceae family have been reported to produce succinate [11, 12]. Several attempts have been made to fight and suppress colon cancer through dietary modifications by some nutritional alternatives in the colon lumen, mainly probiotics (live valuable microorganisms with the potential to enhance microbial balance in the host), prebiotics (nondigestible oligosaccharides), and synbiotics (probiotics and prebiotics combination). Different in vitro, animal, and clinical trials have indicated that probiotics as microbiota modulators and immune response regulators have antitumor efficacy with diverse mechanisms such as the competitive removal of pathogenic intestinal bacteria, enzyme activity alteration of intestinal microflora, decrease in carcinogenic secondary bile acids, attenuation of carcinogens and mutagens binding, and increasing SCFAs production [13–16]. In addition, reduced DNA damage and suppression of ACF development have been thoroughly demonstrated as direct and ideal anti-CRC effects of probiotics in the intestinal mucosa [7, 17].

Therefore, the goal of this study is to evaluate and collect high-quality preclinical studies through a systematic assessment to determine the safety and effectiveness of pro/synbiotics in CRC animal models. Indeed, we performed this systematic review on the studies that explicitly assessed the efficacy of probiotics on gut microbial (bacterial) community composition in CRC animal models and the effectiveness of probiotic supplements on proinflammatory marker alteration and SCFAs production was evaluated.

## 2. Methods

### 2.1. Guidelines

The guidelines defined by PRISMA were followed for this systematic review (Preferred Reporting Items for Systematic Reviews and Meta-Analysis) [18].

### 2.2. Literature Search Strategy

**Figure 1:**
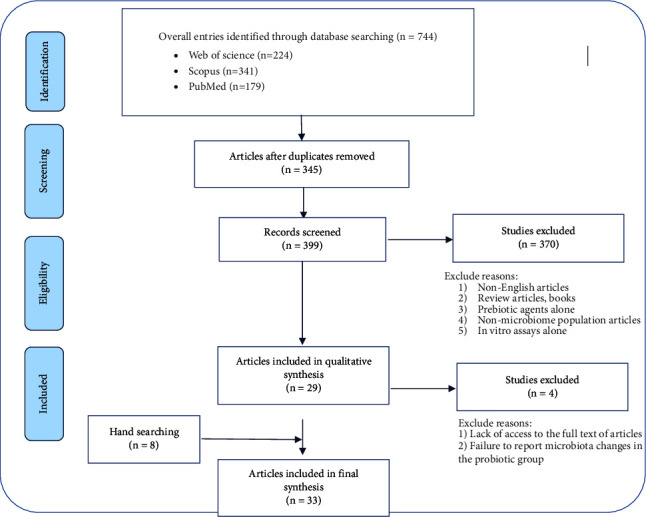
Search and inclusion process of PRISMA flow chart of studies to include in this systematic review.

A systematic search was conducted to evaluate the efficacy of probiotics in the CRC animal models with a focus on microbiota bacterial population. Original research papers were searched in three electronic databases (Scopus, Web of Science, and PubMed) up to December 22, 2021, by two researchers independently. Also, a search was undertaken for grey literature in Google Scholar. Besides, reference lists of all related systematic reviews and articles were monitored to eliminate any potential flaws in online databases and search engines. Initial searches were conducted using terms chosen from our research questions. The terms were “probiotics,” “prebiotics,” “synbiotics,” “colorectal cancer,” “colon cancer,” “CRC,” and also “mice,” “rat,” and “animal model” were searched to improve the results. The search strategy of each database was matched to its particularities. The reference lists of all articles included in this study were also checked for any relevant or ignored studies. After the initial search, two investigators screened the titles and abstracts and excluded articles that did not meet the inclusion criteria. If duplication of the same study was found, its data were included just once. And also, by manually searching Iranian medical laboratory websites, additional relevant papers were discovered and included in a standardized data extraction form. A third investigator double-checked the results to ensure that all eligible articles were considered. Eventually, an alert was set up based on our research keywords in all databases. The flow diagram of the article selection process is represented in Figure 1.

### 2.3. Eligibility Criteria

Studies included in the study met the criteria of being original studies in the animal CRC models, published in English and concerning the administration of probiotics and synbiotics in animal models with CRC. Non-English papers and nonoriginal articles (reviews, editorials, letters, congress papers, comments, abstracts without full text, and book chapters) were excluded. We had limited access to the Embase database, as well as studies that did not meet the eligibility criteria, i.e., (a) studies that used only prebiotics agents; (b) in vitro assays; and (c) clinical studies; and after excluding these items, specifically, studies evaluating microbiota populations were finalized. So, 33 studies that assessed bacterial microbiome compositions by three methods, including high-throughput sequencing, the PCR-denaturing gradient gel electrophoresis (DGGE) fingerprint method, and real-time PCR, were reviewed.

### 2.4. Data Extraction

Two reviewers independently coded and extracted the data from the 33 selected studies. Again, this process was supervised by a third researcher. The data extracted included the following: first author (year of publication), location, animals, number of animals (age range), probiotic types (strain type, doses), interventions, cancer agent and study duration, number of ACF, bacterial profile in gut microbiota, inflammatory markers, SCFAs, and fecal enzymes.

### 2.5. Risk of Bias Assessment

**Figure 2:**
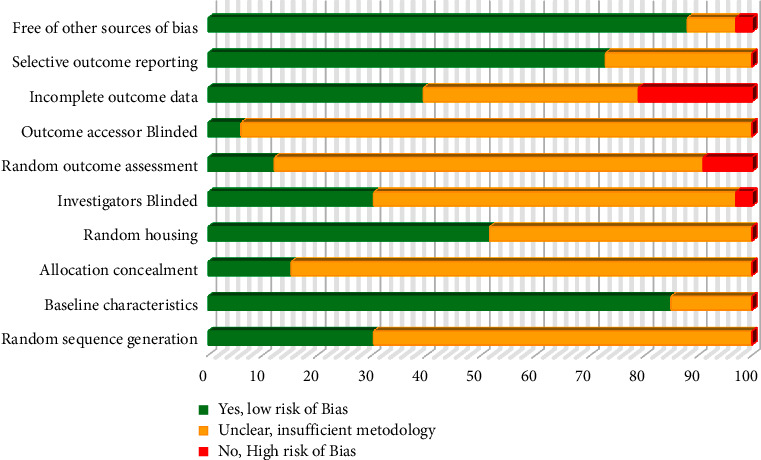
Risk of bias graph displaying each risk of bias item presented as percentages across all 33 studies. The 10 signaling questions of the SYRCLE's risk of bias assessment tool were used. A “Yes” indicates a low risk of bias, a “No” indicates a high risk of bias, and an “Unclear” indicates that insufficient methodology.

The risk of bias (RoB) in animal studies was assessed using the SYRCLE's (Systematic Review Center for Laboratory animal Experimentation) tool [19]. This instrument is based on the Cochrane Collaboration's tool for assessing the risk of bias in randomized trials and is modified for biases specific to animal intervention studies [19]. The following methodological parameters were evaluated using standardized questions to guide the researcher's judgment: Selection bias: “Sequence generation,” “Baseline characteristics,” “Allocation concealment”; Performance bias: “random housing” and “blinding of investigators regarding the intervention that each animal received during the experiment?”; Detection bias: “random outcome assessment,” “Was the outcome evaluator-blinded?”; Attrition bias: “Incomplete Outcome Data”; Reporting bias: “Is there any selective outcome reporting in the study's reports?”; Other biases: “Other potential sources of bias that could lead to a high risk of bias.” The items in the RoB tool were graded with “Yes” (low risk of bias); “No” (high risk of bias); or “Unclear” (the item was not reported and or insufficient information and methodology; consequently, the risk of bias was unknown) (Figure 2).

## 3. Results

### 3.1. Literature Search and Study Selection

The search strategy submitted a total of 744 papers in the following databases: Scopus, Web of Science, PubMed, and Google Scholar. The process of study selection is shown in the PRISMA flowchart (Figure 1). In the second screening phase, 345 duplicate publications were removed, and 399 articles were retained for detailed full-text evaluation. Three hundred and seventy articles were excluded for the following reasons: articles were not original studies (comments, books, editorials, and reviews), there were no English language papers, studies of microbiome compositions were not evaluated; prebiotics were employed alone against CRC, unrelated cancers, and in vitro models alone. Four out of the 29 studies were removed due to the lack of full text (2 articles), and in two articles, there was no mention of the bacterial profile composition in probiotic groups. On the other hand, eight papers were added to our study by hand searching. Eventually, 33 articles describing the efficacy of the probiotics on CRC treatment in animal models, especially based on evaluating the microbiome population, were included in our analysis.

### 3.2. Characteristics of the Included Studies

The summarized characteristics of 33 studies are presented in Table 1. These studies were published from 1/1/2010 to 12/14/2021. The selected studies were performed in 13 different countries. The majority of the studies were conducted in China (16 out of 33 studies) [20–30]. Among the 33 studies, 28 trials used only probiotics (single or in combination with other probiotics) to investigate their impact on colon cancer [20, 21, 23–48]. Five trials used prebiotics as an intervention agent in combination with probiotics [22, 49–52]. Besides, as shown in Table 1, among the 33 studies, 8 studies used a combination of multistrain probiotic bacteria [22, 26, 28, 30, 37, 44, 46, 52]. In these 8 studies, *L. acidophilus* (4 studies) showed the highest number of combinations with other bacteria [22, 26, 37, 46]. Probiotic administration in a total of 33 studies was oral because it is safer, cheaper, and more controllable.

**Figure 3:**
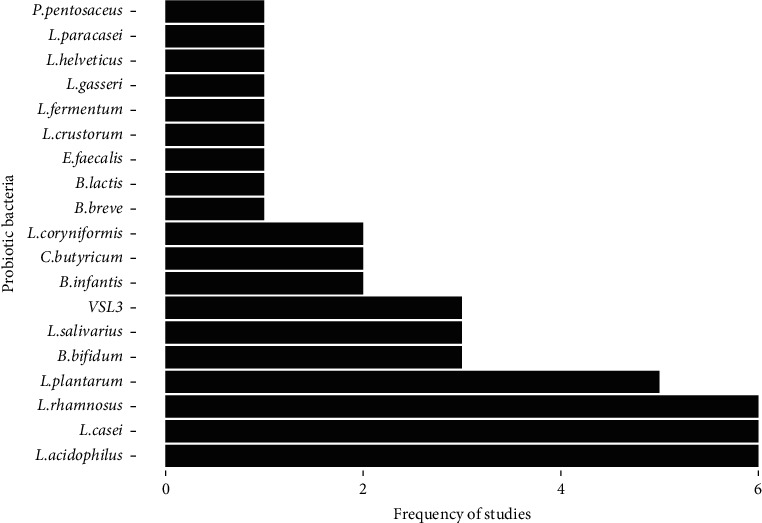
The frequency of probiotic species used to treat CRC animal models in 33 studies.

A total of 19 different probiotic species were administered daily at doses of 1 × 10^7^ to 6.4 × 10^11^ colony forming units (CFU) alone and/or in combination with each other. Based on Figure 3, *L. acidophilus, L. casei,* and *L. rhamnosus* (18.2%) were the most common probiotics used by the included studies.

In these studies, some unique probiotics such as *C. butyricum* (2 trials) [20, 23], *E. fecalis* (1 study) [26], *P. pentosaceus* (1 trial) [40], and Kefir (1 trial) [43] were used as probiotic agents in CRC models. In 33 included preclinical studies, the major models used were mice (25 studies) [20, 23–31, 33, 35, 36, 38–40, 42, 45–52], the age of the animals ranged from 3 to 16 weeks, and the duration of the study was 28 days to 32 weeks. Among 33 articles included, the most prominent cancer agent was azoxymethane/dextran sodium sulfate (AOM/DSS) (14 studies) [23, 25–29, 33, 35, 36, 39, 40, 45, 46, 48]. The second most commonly used cancer agent was 1,2-dimethylhydrazine (DMH) as the inducing agent of preneoplastic lesions and tumors were induced, and the doses varied from 10 to 40 mg/kg (11 studies) [21, 22, 32, 34, 37, 41–43, 49–51]. In 3 studies, genetically modified animals, in which disease developed spontaneously, were used [20, 24, 31]. In addition, in 2 studies, CT-26 cell lines were used as cancer causes [30, 38]. In 2 trials, colorectal tumors were induced in animals by cyclic treatment with dextran sulfate sodium (DSS) [44, 52]. Finally, one study did not mention cancer agents [47].

### 3.3. Effects of Probiotics on Histopathological Characteristics

#### 3.3.1. Analysis of Aberrant Crypt Foci (ACF) in Dealing with Probiotics

Five studies presented the results of the development of ACF after the animals were exposed to the carcinogen, and ACF was identified and analyzed by methylene blue staining in all studies [21, 34, 43, 49, 50]. ACF is recognized as a precancerous lesion of CRC (preneoplastic lesions) that persists, grows in the distal colon, and has the potential to progress to cancerous tissue. In these five references, probiotics alone or combined with prebiotics effectively reduced colon ACF incidence and multiplicity in the CRC models. In 2 studies [21, 34], *L. salivarius* [34] and *L. rhamnosus* [21] treatments showed a significant decrease in total ACF number compared with DMH-treated rats. Ali et al. [49] demonstrated the administration of *L. casei*, inulin, and synbiotic (*L. casei* + inulin) significantly decreased the number of ACF by three, five, and six times, respectively, compared to the DMH-treated group (*p* < 0.001). Besides, Cruz et al. [50] demonstrated synbiotic (yacon diet + *VSL#*3) consumption reduced the incidence of total ACF by 38.1% compared to the DMH group (*p*=0.001). Interestingly, de Almeida Brasiel et al. [43] reported that intake of Kefir as potential probiotic fermented milk had no significant effect on the number of ACF between cancerous groups and Kefir-treated cancerous groups.

#### 3.3.2. Epithelial Proliferation Changes in Probiotic Treated Animals

In six studies [20, 23, 25, 38, 45, 48], epithelial proliferation was assessed by Ki67 staining as a cell proliferation marker protein. The proliferation index was determined by counting the number of Ki67-positive cells per crypt. In 5 studies [20, 23, 25, 45, 48], probiotics significantly suppressed the proliferating cells per crypt in the colons of probiotic-treated mice. In one study by Chang et al. [38], *Lactobacillus casei* variety *rhamnosus* (*Lcr*35) supplementation did not affect intestinal crypt proliferative activity. Besides, Zhu et al. [34] evaluated colonic cell proliferation in rats by PCNA staining. They showed that *Lactobacillus salivarius* Ren suppressed the cell proliferation of colonic mucosa in cancerous rats.

#### 3.3.3. Effect of Probiotic Treatment on Goblet Cell Percentages

In four studies [28, 29, 38, 49], the number of goblet cells was analyzed. In 3 studies, probiotics [28, 29] and synbiotic [49] administration prevented AOM/DSS and DMH-induced goblet cell loss in CRC models. On the other hand, Chang et al. demonstrated that *Lcr*35 at the highest dose did not significantly reduce goblet cell damage in the cancerous group [38].

#### 3.3.4. Effects of Probiotics on Gut Barrier Integrity in CRC Models

The integrity of the gut mucosa and the intestinal epithelial barrier was examined by mucin-2(MUC2), adherence junction protein-1 (ZO-1), and tight junction (occludin) proteins in 4 studies [22, 28, 29, 51]. They found that the expression of these proteins declined significantly in the AOM/DSS [28, 29] and DMH [22, 51]-induced mice, and these alterations were dramatically reversed in cancerous animals treated with probiotics and synbiotics.

#### 3.3.5. Effect of Probiotics on Inflammatory Cell Infiltration in the Colon

Hematoxylin and Eosin (H & E) staining results from 8 studies [23, 28, 29, 31, 43, 44, 46, 52] revealed that probiotics and synbiotics administration significantly reduced the degree of inflammatory cell infiltration and crypt damage in CRC animal models.

Altogether, the major effects of various probiotics on the development of histopathological parameters in CRC models were collected in this study including (1) Probiotic application alone or with prebiotics limited the development and incidence of ACF as preneoplastic lesions. (2) Probiotics suppressed the proliferation of intestinal tumor cells and upregulated their apoptosis. (3) Probiotics alone or in combination with prebiotics could help to recover and prevent goblet cell loss caused by CRC. (4) Probiotic and synbiotic administration ameliorated intestinal gut barrier integrity by enhancing some related proteins in CRC cancerous models. (5) Probiotic alone or with prebiotics moderated inflammatory cell infiltration in CRC animals.

### 3.4. Efficacy of Probiotics on Shifts in Fecal Microbiota Compositions

In Table 2, we summarized and identified the impact of probiotics and synbiotics on the relative distribution of bacterial communities in the gut microbiome at the phylum, family, genus, and species levels between cancer groups and probiotic/synbiotic-treated cancer groups.

#### 3.4.1. Phylum Level

Based on Table 2, among 33 reviewed studies, the major predominant phyla were *Bacteroidetes* (10 studies)*, Firmicutes* (9 studies)*, Proteobacteria* (8 studies)*, Verrucomicrobia* (5 studies)*, Actinobacteria* (4 studies), and *Deferribacteres* (3 studies), respectively. The abundance of phylum *Bacteroidetes* decreased in 7 studies: 3 studies by synbiotics [22, 50, 51] and 4 studies by probiotics [27, 36, 39, 47]. *Bacteroidetes* increased in 3 studies [23, 33, 38] (2 studies by probiotics [23, 33], and one study by synbiotic [38]). And also, *Firmicutes* were assessed in 9 studies (out of the 33 studies) [20, 23, 27, 31, 38, 45, 47, 50, 51]. In 4 studies (4 out of the 33) [23, 38, 50, 51], *Firmicutes* declined significantly in treated CRC animals (2 studies by probiotics [23, 38] and 2 studies by synbiotics [50, 51]). But in 3 other studies, the *Firmicutes* level had an increasing trend by probiotic agents alone [31, 45, 47]. In Chen et al.'s study [20], *Firmicutes/Bacteroides* ratio was insignificant by *C. butyricum* in Apc^min/+^ mice. Interestingly, in Cruz et al.'s study [50], *Firmicutes* level was increased by *VSL*#3 while it had a declining trend in the synbiotic + DMH animal group.

In 24% of studies (8 out of the 33 studies) that detected *Proteobacteria* in phylum level [22, 27, 28, 35, 36, 47, 50, 51], four studies identified an increment in treated CRC models (3 studies by probiotics (27, 28, 36) and one study by synbiotic [51]). In contrast, four articles showed a significant decreasing trend in the phylum of *Proteobacteria* in probiotics (2 studies [35, 47]) and synbiotics (2 studies [22, 50]) treated CRC animals. In addition, *Verrucomicrobia* as one of the most prevalent phyla in the gut microbiome was shown in 15% of studies (5 out of the 33 studies) [27, 33, 36, 47, 49]. In 3 studies, the prevalence of this phylum decreased notably in probiotic-treated cancerous models [33, 36, 47], While it developed in 2 studies by *B. bifidum* [27] and synbiotic [49]. Finally, *Actinobacteria* in 12% of studies (4 out of the 33 studies) [27, 31, 46, 51] and *Deferribacteres* in 9% of studies (3 out of 33 studies) [25, 26, 33] were identified in the phylum level.

#### 3.4.2. Family Level

At the family level, Lachnospiraceae and Ruminococcaceae (in 5 studies) [20, 23, 27, 40, 50, 51] were reported as the main families among 33 included studies. In 2 studies by Wang et al. [27] and Chung et al. [40], the abundance of these two bacteria simultaneously in the family level increased dramatically by probiotic agents in cancer groups. Interestingly, dos Santos Cruz et al. [51] determined that Lachnospiraceae and Ruminococcaceae declined together by synbiotic (*VSL*#3 + PBY) intervention in CRC animals. In another study by Cruz et al. [50], the abundance of Lachnospiraceae at the family stage was raised in CRC models that received *VSL*#3 + Yacon as synbiotic agent. Surprisingly, *C. butyricum* had different effects on the abundance of the two families mentioned above. Liu et al. [23] showed that Lachnospiraceae decreased in *C*. *butyricum-*treated cancerous animals. On the other hand, in Chen et al.'s study [20], Ruminococcaceae were increased by treatment with *C*. *butyricum* in CRC groups. Furthermore, *Lactobacillaceae* had significant expansion in 3 studies [21, 40, 50] by *P*. *pentosaceus* [40], *VSL*#3 + Yacon [50], and *LGG* [21] in cancer groups. And also, Prevotellaceae were reported in 3 studies [21, 25, 33], with an increment in 2 studies by *L*. *casei* [33] and *LGG* [21] and a reduction by *L*. *helveticus* [25] in cancerous animals. Besides, the Porphyromonadaceae level was reduced in 2 studies by *L*. *helveticus* [25] and *VSL*# [36] in CRC involving animals. Moreover, Bacteriodaceae [21, 25] and Clostridiaceae [21, 50] were reported in two studies with an increasing trend in probiotic and synbiotic-treated CRC models. Eventually, in one study, Akkermansiaceae, as essential beneficial probiotic bacteria, were significantly enriched in response to the treatment of *P*. *pentosaceus* in CRC models [40].

#### 3.4.3. Genus Level

At the genus level, in 13 out of 33 studies [21–23, 26–28, 39, 43–47, 50], *Lactobacillus* was the most predominant genus reported among included studies and improved notably by probiotic and synbiotic agents in CRC animal groups, and then *Akkermansia* as one of the main genera was discovered in 7 studies (7 out of 33 studies) [24, 27, 29, 39, 46, 48, 49]. Five out of 7 studies showed that this genus increased remarkably in probiotics-treated cancerous animal groups. But, in 2 studies, the *Akkermansia* genus declined in *L*. *fermentum* [39] and *LGG* [24] in groups challenged with CRC. Besides, *Prevotella* as butyric acid-producing bacteria at the genus level had a remarkable increase in 3 studies in probiotics-treated CRC-models [23, 30, 45]. But Rong et al. [25] and de Almeida Brasiel et al. [43] reported that the shifts towards the increased abundance of *Prevotella* in mice with colitis and tumors were lowered by *L*. *helveticus NS8* and Kefir as probiotic agents. And also, *Turicibacteria* was shown in 5 studies [23, 27, 28, 42, 49]. In 4 studies [23, 27, 42, 49], it was increased notably by probiotics and synbiotics. In contrast, in one study by Wang et al. [28], the abundance of these bacteria decreased with a mixture of probiotics in CRC animal groups. Another dominant genus was *Desulfovibrio* which was reported in 5 trials [20, 26, 28–30], and in all studies, it was decreased significantly by probiotics agents in CRC groups. As well, the *Helicobacter* genus (in 4 out of 33 studies) had a contraction trend in probiotic and synbiotic-treated groups [20, 22, 29, 50]. Finally, *Roseburia* was reported in 4 studies with an increased trend by probiotics and synbiotics in CRC models [24, 28, 30, 50]. Totally at the genus level, probiotics exhibited a superior protective effect against CRC induction agents by enriching beneficial bacteria in the colon, such as *Lactobacillus*, *Akkermansia*, *Prevotella*, *Turicibacteria*, and *Roseburia*.

#### 3.4.4. Species Level

Different *Bacteroides* spp. were identified in 5 studies [25, 30, 32, 33, 37]. According to Rong et al.'s study [25], *Bacteroides acidifaciens* increased and *Bacteroides uniformis* decreased by *L*. *helveticus NS8* in groups in which AOM/DSS was inoculated into animals as a carcinogen. In addition, *Lactobacillus* spp. increased significantly in 5 studies [25, 37, 39, 45, 52] by probiotics alone or a mixture of probiotics. Finally, in 2 studies, different species of *Bifidobacterium* had an increment trend in *Lactobacillus* alone or mixed with other probiotics in cancerous models [25, 37].

### 3.5. Effects of Probiotics and Synbiotics on the Fecal Concentration of SCFA

Based on Table 2, in 10 studies, production of SCFAs along with microbiota composition was assessed [20, 24, 28, 29, 33, 34, 42, 44, 50, 51]. Seven studies showed that high levels of SCFAs secretion were observed in cancer groups receiving different species of *Lactobacillus* alone or in mixtures with *Bifidobacterium* [24, 28, 29, 33, 34, 42, 44], and Chen et al. [20] reported that the levels of acetic, propionic, and butyric acid (*P* < 0.001) in the *C. butyricum*-treated cancerous mice were significantly higher than in cancerous groups. Moreover, two studies by Cruz et al. [50, 51] revealed that synbiotic-treated animals had higher concentrations of acetic, propionic, and butyric acids at all times.

Besides, only in one study [20], the levels of the fecal secondary bile acids (BAs) DCA and LCA were evaluated, and the primary BAs were not affected by *C*. *butyricum* treatment. The levels of the fecal secondary BAs DCA and LCA were markedly increased in the HFD-treated mice compared with the control (DCA: *P* < 0.001; LCA: *P* < 0.01). Surprisingly, the DCA and LCA levels in the *C. butyricum* group decreased significantly (DCA: *P* < 0.05; LCA: *P* < 0.01).

### 3.6. Fecal Enzymes Assay

Fecal enzymes, i.e., *β*-glucuronidase, azoreductase, and *β*-glucosidase have been implicated in converting procarcinogens into carcinogens; thus, the activity of these enzymes was assessed to deduce the modulating potential of probiotics in the colonic environment. Only three studies [34, 41, 50] reported changes in fecal enzyme activity in probiotic and synbiotic-treated groups (Table 2). In 2 studies, fecal enzymes were assessed along with bacterial microbiota compositions and SCFAs [34, 50]. Also, Zhu et al. [34] revealed that there was a significant decrease in azoreductase activity in LS-treated + DMH rats when compared to DMH rats, while the activities of *β*-glucosidase and *β*-glucuronidase were not significantly affected by LS treatment. Moreover, CRC animals receiving the synbiotic displayed a significant reduction in *β*-glucuronidase enzyme activity when compared to the control group. In addition, in both studies, SCFAs production was increased notably by probiotics and synbiotics.

### 3.7. The Effect of Probiotics on Inflammatory Marker Expression

**Figure 4:**
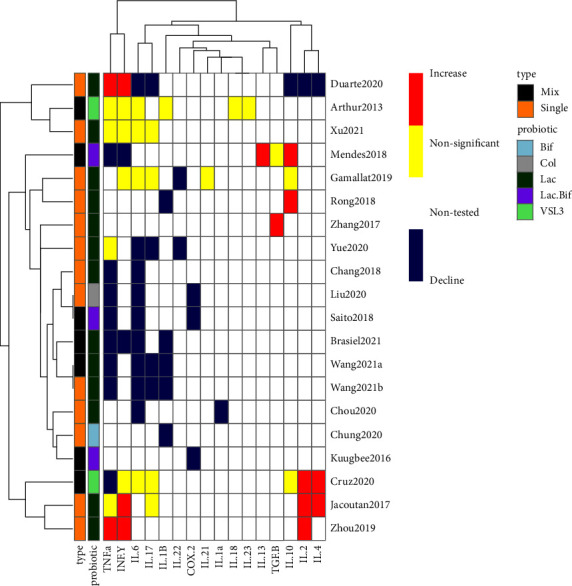
Heat map analysis of probiotics impact on inflammatory markers expression in CRC models. Inflammatory marker levels were compared between probiotic and/or synbiotic treated cancer groups and cancer groups alone (without treatment) in 20 studies. Bif: *Bifidobacterium*; Clo: *Clostridium*; Lac: *Lactobacillus*; Lac. Bif: *Lactobacillus Bifidobacterium*; Mix: mix of probiotics; Single: single probiotic.

The effects of probiotics on inflammatory marker expression were assessed in 20 studies [21–23, 25, 28, 29, 31, 33, 35, 36, 38–40, 42, 43, 45, 46, 48, 50, 52] (Figure 4). In these studies, 16 cytokines were assessed and quantified by western blotting, real-time PCR, and ELISA methods. Fourteen out of 20 studies (70% of preclinical trials) [23, 28, 29, 31, 35, 36, 38, 42, 43, 45, 46, 48, 50, 52] assessed TNF-*α* levels in cancer models that received probiotics, and in 57% of the included studies (8 out of 14 studies) [23, 28, 29, 38, 43, 46, 50, 52], TNF-*α* declined significantly in probiotic-treated groups. Besides, among the 20 preclinical trials, 13 trials (65% of studies) [21, 23, 28, 29, 31, 36, 38, 39, 42, 43, 48, 50, 52] analyzed the effects of probiotics on IL-6 levels. In 9 out of 13 studies (69% studies) [23, 28, 29, 31, 38, 39, 42, 43, 52], IL-6 levels decreased with probiotics treatment in CRC animal groups. While in 30% of studies (4 out of 13 studies) [21, 36, 48, 50], IL-6 changes were nonsignificant. The next prominent marker (INF-*ɣ*) was investigated in 9 out of 20 studies (45% of studies) [21, 35, 36, 42, 43, 45, 46, 48, 50]. The expression of this marker was nonsignificant in 44% of included studies [21, 36, 48, 50] and increased in 33% of studies [35, 42, 45] by probiotic agents in included studies. In 8 out of 20 studies (40% of trials) [21, 28, 29, 31, 42, 45, 48, 50], IL-17 expression was detected, and 50% of the included studies (4 out of 8 studies) [28, 29, 31, 42] showed a decrease trend in the probiotic-receiving group. But in 50% of studies, this change was unnoticeable [21, 45, 48, 50]. Moreover, the effect of probiotics on IL-1*β* levels was detected in 6 out of 20 studies (30% of included studies) [25, 28, 29, 36, 40, 43], and in 83% of included studies (5 out of 6 studies) [25, 28, 29, 40, 43], this marker declined significantly by probiotic agents. Finally, each of the following cytokines, including IL-1*α* [39], IL-13 [46], IL-18 [36], IL-21 [21], and IL-23 [36], was reported only in one study with no significant changes in probiotic treatment groups.

### 3.8. The Regulation of Signaling Pathways in Dealing with Probiotics

The effects of probiotics/synbiotics on the regulation of apoptotic markers were measured in 11 studies [20–23, 25, 31, 33, 38, 42, 45, 49] (Table 2). In these studies, NF-*κ*B, Cox-2, Bcl-2, and *β*-catenin expressions were elevated in cancerous groups but depleted in probiotic and synbiotic-treated CRC groups. In two studies, da Silva Daurte et al. [42] and Gamallat et al. [21] found that LGG + DMH-treated animals had higher p53 expression (classic tumor suppressor gene) than DMH-treated animals. In three studies [21, 23, 38], probiotics administration reduced Bcl-2 expression while increasing Bax expression, demonstrating that probiotics could inhibit colorectal cancer development in animals by promoting the expression of proapoptotic genes.

### 3.9. Risk of Bias Assessment

The SYRCLE's tool [19] was used to assess the methodological quality and potential risk of bias in the 33 included studies (Figure 2). Compared to randomized clinical trials, poor reporting in preclinical studies is a known issue. In this systematic review, the majority of preclinical trials showed a questionable risk of bias (unclear) in aspects such as describing their randomization, blinding process, and allocation process. Only two studies (6% of studies) mentioned blinding outcome assessors. The highest risk of bias was shown in the incomplete outcome data items (21% of studies). Most articles (88% of studies) were found free of other sources of bias. None of the articles were considered to show selective reporting. Consequently, the majority of assessments were scored as unclear risk of bias (53% of studies) and then low risk of bias (43% of studies) in the current 33 studies.

## 4. Discussion

**Figure 5:**
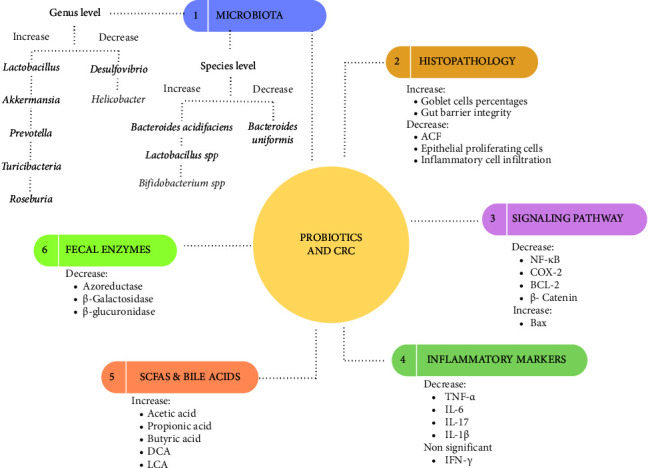
The summary of probiotic potential mechanisms on CRC. The rates of increase and decrease in the microbiota composition and inflammatory markers sections are based on the majority of the articles, not all of them. This diagram is drawn in the Canva web application and the following link provides you with its editable version: https://www.canva.com/design/DAFmjVgqkP0/l-7GhS3AbVkJFG8baGjFmQ/edit?utm_content=DAFmjVgqkP0&utm_campaign=designshare&utm_medium=link2&utm_source=sharebutton.

CRC is the third most prevalent cancer in both men and women, and most of its environmental etiological factors, such as changes in dietary habits and lifestyles, can be regulated therapeutically to minimize the risk of disease development. Regular intake of probiotics would present a more effective anticarcinogenic effect in the early stages of CRC [53]. In the current systematic review (data analysis from 33 studies), we collected the outcomes of the effect of various probiotic strains alone or in combination with prebiotic supplements (as a synbiotic) on ACF incidence, gut bacterial populations, levels of inflammatory markers, and SCFAs metabolites levels with fecal enzyme secretion in CRC preclinical models (all factors were summarized in Figure 5).

ACF is recognized as a CRC precancerous lesion, and significant numbers of ACF are detected in full-blown CRC models. Increased ACF numbers are associated with a higher risk of CRC [54]. Based on five studies in the current review, the number and percentage of ACF decreased notably in CRC models that received probiotics [21, 34, 43, 49, 50]. And also, Cruz et al. [50] demonstrated that synbiotics administration reduced the percentage of ACF occurrence (38.1%) more than probiotic supplementation (19.8%) compared to the cancer group. Indeed, synbiotics were more effective than probiotics at reducing ACF development. The possible mechanisms of ACF failure by pro/synbiotics in CRC animals can be the following: (a) Prior pro/synbiotics supplementation (before induction of cancer agents) [55]. (b) Preventing DNA damage in the colon through the complex interaction of probiotics or their metabolites with cancer metabolites by increasing the number of fecal lactobacilli in the gut microbiome which may have inactivated some procarcinogenic enzymes such as *β*-glucuronidase, nitroreductase, and *β*-glucosidase enzymes, resulting in lower ACF counts [56, 57]. (c) Brief adherence of probiotics to the colonic epithelial cells may protect the epithelial barrier from carcinogens and their metabolites while also reducing the binding and contact time of epithelial cells to carcinogens [58, 59]. (d) A positive correlation between ACF incidence and decreasing fecal pH (acidification of the colonic content) by SCFAs produced by probiotics [50]. (e) High-level secretion of IFN-*γ* as an antiproliferative and antiangiogenic protein by some probiotics and its noticeable apoptotic activity, which may diminish ACF [21]. It is worth noting that the observed difference in percentages of ACF reduction by different probiotics could be attributed to the fact that probiotic response is species- and strain-specific.

In this systematic review, we have focused on the association between probiotic/synbiotic supplementation and the regulation of gut microbiota bacterial profiles in CRC animal models. *Lactobacillus* and *Bifidobacterium* were the most widely investigated probiotic bacteria, followed by *C*. *butyricum* (2 studies) and *P*. *pentosaceus* (1 study). These reviewed studies have reported that probiotic intervention (single/mix) or along with prebiotics (synbiotic) markedly improved the abundance of microbiome-friendly bacteria such as *Lactobacillus*, *Bifidobacterium*, *Akkermansia*, *Romboutsia*, and *Roseburia* in the preclinical CRC context. Besides, based on ten studies [20, 24, 28, 29, 33, 34, 42, 44, 50, 51] in this review that assessed SCFAs along with bacterial communities in microbiota, SCFAs-producing bacteria showed an increasing trend along with higher production of SCFAs in pro/synbiotic-treated groups. These bacteria included the following: (1) *Turicibacter* has been linked directly to the production of butyric acid, which acts as an immunomodulator with anti-inflammatory activity in Cruz et al. and da Silva Duarte et al.'s studies [42, 50]. (2) Increased abundance of *Roseburia* as a butyrate and short-chain fatty acid-producing bacteria, along with high butyric acid levels in 3 studies [24, 28, 50]. (3) Increasing prevalence of *Clostridium XI* and *Clostridium XVII* (as important butyrate producers) [20] after probiotic/synbiotic supplementation with high levels of butyric acid production in CRC models [34, 50]. (4) High proportion of Lachnospiraceae (SCFAs producing bacteria) in family, genus, and species levels along with an increase in butyrate in Zhu et al. [34], Wang et al. [28], and Cruz et al.'s [50] studies. (5) Greater relative abundance of *Prevotella* and *Alloprevotella* from the Prevotellaceae family with higher butyric acid levels [33, 34]. (6) *Ruminococcaceae* and *Eubacterium*, which are well known to produce SCFAs, were elevated together with total SCFAs in a study by Chen et al. [20]. (7) Enrichment of *Lactobacillus*, *Bifdobacterium*, *Akkermansia*, and *Faecalibaculum* that is produced simultaneously by *L*. *coryniformis* MXJ32 with high levels of SCFAs [29]. Besides, treatment with a probiotics mixture showed a significant increase in some SCFAs-producing bacteria simultaneously, including Lachnospiraceae*_NK4A136*_group, *Faecalibaculum*, *Roseburia*, and *lactobacilli* in 2 studies [28, 44]. Indeed, SCFAs as beneficial metabolites of gut microbiota have multiple pathways to ameliorate CRC, including suppressing bacterial pathogens [60], regulating cell proliferation and differentiation [61], preserving colonic epithelial health, reducing inflammation, and inhibiting histone deacetylases[62]. Butyrate-producing bacteria, in particular, are more prominent since butyrate acts as a histone deacetylase inhibitor, modulating the expression of oncogenes and boosting the secretion of anti-inflammatory cytokines [63]. However, it is worth noting that some of these beneficial bacteria in response to probiotics showed contradictory manners in current reviewed studies. For example, at the family level, Lachnospiraceae and Ruminococcaceae as SCFAs producing bacteria decreased dramatically in two studies [23, 51] in response to pro/synbiotics. Besides, *Turicibacter,* surprisingly, showed a negative correlation with butyrate in Wang et al.'s study [28]. Also, the *Akkermensia* genus, as a type of Gram-negative bacteria and a promising probiotic candidate, declined in two studies [24, 39] in response to probiotic treatment in cancerous groups.

Furthermore, *β*-glucuronidase, *β*-glucosidase, and azoreductase bacterial enzymes are associated with the conversion of procarcinogens to potential carcinogens in the colon by the release of cytotoxic and genotoxic metabolites [14, 64]. Modulating the activity of these bacterial enzymes could be one of the mechanisms by which probiotics could minimize exposure to carcinogenic substances and hence reduce colorectal cancer development. For instance, the genera *Bifidobacterium* and *Lactobacillus* displayed minimal *β*-glucuronidase activity for modifying the microbiota [65]. In addition, based on two studies [41, 50] in our review, *β*-glucuronidase activity decreased in response to probiotic and synbiotic supplementation. While Zhu et al. [34] identified *β*-glucosidase and *β*-glucuronidase showed no alterations in *L*. *salivarius*-treated cancerous animals, still azoreductase activity was reduced in this group, indicating a possible protective effect against carcinogenesis by decreasing levels of carcinogen activation and DNA mutation.

Based on 33 reviewed papers, it was demonstrated that many immune pathways inhibit carcinogens by probiotics in animal CRC models that these potential and diverse pathways briefly include the following:

1. Protective effects of pro/synbiotic against colon cancer through increasing the phosphorylated JNK-1 expression as well as boosting beneficial bacteria in the colon such as *Akkermansia* and *Turicibacter* while decreasing the expression of phosphorylated GSK3b and *β*-catenin [49].
2. Probiotics might be able to suppress inflammation and improve mucositis in the intestine by inhibiting NF-B activity (which upregulates proinflammatory cytokines TNF-, IL-1, and IL-6), and TNF- and IL-6 proinflammatory effects could be reduced by taking probiotics [38].
3. Reducing pathogenic bacteria and infiltration of CD68^+^ macrophages (limiting macrophages recruitment) and then a significant reduction in proinflammatory markers such as IL-1*α*, IL-1*β*, and IL-6 by probiotics [39].
4. A marked increase in IL-2 and IL-4 by probiotic and synbiotic in CRC models which results in the regulation of immune cells and antitumor defense [50, 66].
5. The increased IFN-*γ* protein secretion by *LGG* (probiotic) in CRC animals is consistent with the considerable apoptotic activity, immune-regulatory function, antiproliferative, and anticancer strategies of IFN-*γ* [21].
6. Probiotics induce apoptosis in CRC tumors by significantly decreasing Bcl-2 levels while increasing Bax, caspase-3, and p53 expression. In addition, probiotics block Cox-2, which activates the downstream target proapoptotic protein p53, which links to tumor suppression [21, 67, 68].
7. Inhibitory effect of probiotics on *Bacteroides* that releases metalloprotease and fragilysin toxins which help CRC promotion through boosting IL-17a production and inducing E-cadherin cleavage [21, 69].
8. Beneficial effects of a strain of *Lactobacillus reuteri* in a model of CRC by a histidine decarboxylase (HDC), which downregulated IL-22 expression levels that were enhanced in tumor tissues [45, 70].
9. Attenuating the over-activation of TLR4/NF-*κ*B in the CRC models by probiotics and inhibiting inflammation by preventing the release of certain reinflammatory cytokines (TNF-*α*, IL-1*β*, and IL-6) and CXCR2 ligand chemokines (CXCL1, CXCL2, CXCL3, CXCL5, and CCL7) [29].
10. Great probiotic potential and anti-inflammatory effect of *Lactobacillus* by downregulating proinflammatory markers such as IL-6, IL-17 F, and IL-22 [31, 71, 72].

It is worth mentioning that none of the concluded studies reported a reverse or negative effect of probiotics against CRC in animal models except the Arthur et al.'s study [36]. In this study, an unexpected result of consuming *VSL*#3 probiotic was the lack of an inhibitory effect on tumorigenesis and the tendency to enhance tumor invasion in AOM/Il10^−/−^ mice. They have previously shown that inflammation affected the composition of the intestinal microbiota in Il10^−/−^ mice, leading to an increase in *Proteobacteria*, which influenced CRC formation. In the current study [36], they demonstrated that *VSL*#3 administration after the initiation of inflammation and dysbiosis can boost tumorigenesis and primarily induce the elimination of beneficial bacteria such as *Clostridium*.

Together, these 33 studies have revealed that the anti-CRC effects of probiotics arise through alteration of the composition of the microbiota by various mechanisms, including (i) probiotics promote microbiota hemostasis by competing with putrefactive and harmful bacteria, reducing their abundance while increasing the number of LAB bacteria. (ii) In spite of their strong adhesion to the intestinal epithelium, probiotics are noninvasive and inhibit pathogen adhesion to the intestine [22, 73]. (iii) By lowering the pH of the environment, probiotics inhibit the proliferation of detrimental bacteria, and during this period, beneficial bacteria flourish in the acidic environment, balancing the intestinal microbiota [74]. (iv) Antimicrobial substances produced by probiotics in microbiota include bacteriocins, deconjugated bile acids, reuterin, hydrogen peroxide, and lactic acid, which can be used by probiotic microbiomes as a means of inhibiting pathogenic and carcinogenic bacteria populations [75]. (v) In addition to reestablishing gut microbiota balance, probiotics stimulate the secretion of anti-inflammatory cytokines by regulatory T (Treg) cells and IgA in intestinal epithelial cells and decrease proinflammatory pathways (through decreased levels of IL-1*β*, IL-6, and TNF-*α*). A study by Gao et al. found *Roy*'*s Lactobacillus* can suppress the incidence of inflammation-related CRC in mice by secreting histamine, and this suppresses tumor growth by producing histamine [70]. (vi) Probiotics improve SCFAs as bioactive metabolites of bacteria, regulating gastrointestinal microecology and energy balance as well as the CRC cell proliferation inhibiting through the Wnt/*ß*-catenin pathway. (vii) By modulating the activity of fecal bacterial enzymes such as *β*-glucuronidase, *β*-glucosidase, and nitroreductase, probiotics can significantly change the metabolism structure of detrimental bacteria in the gut microbiota. (viii) Probiotics increase mucin production and tight junction protein expression to improve gut-barrier function.

### 4.1. Strengths and Limitations

Taken together, the remarkable strength of this systematic review is that we have presented all the latest preclinical papers that investigated the potential beneficial effects of probiotics in addressing colorectal cancer animal models by focusing on microbiome bacterial populations. In addition, histopathological changes, signaling pathways, inflammatory markers alteration, short-chain fatty acids, and fecal enzyme release were gathered systematically in these studies. Nevertheless, there are some limitations in our study because of the different methodological designs and protocols of the included studies such as the use of diverse ranges of probiotic strains, prebiotics products, and cancer induction agents, as well as the administration of lots of dosages with different duration of treatment and different pathways for the preparation of probiotic supplements in these studies, which prevented us from drawing absolute conclusions and pooling the included studies in a meta-analysis. Despite this heterogeneity, we tried to collect some homogeneous data based on 33 studies; these include (1) the bacteria which are the most beneficial? Based on Figure 1 and Table 1, 85% of studies used members of the genus *Lactobacillus* alone or in a mixture with other probiotics with strong positive effects on modulation of gut microbiota composition by enriching beneficial bacteria, especially *Lactobacillus*, *Akkermansia*, *Prevotella*, *Turicibacteria*, *Roseburia*, and other markers. (2) the dose of which probiotics administration is the most effective? The analysis of probiotic dosage revealed significant differences among the 33 studies. A total of 19 different probiotic species were administered daily orally at doses of 1 × 10^7^ (in mice) to 6.4 × 10^11^ (in rats) CFU in two best-recommended averages ≥10^9^ and ≥10^8^ CFU, respectively, displaying the best achievement such as improving the abundance of gut microbiota-friendly bacteria. (3) How many weeks was the best time to study? The duration of studies ranged between 4 and 24 weeks in three categories, as follows: (1) study period from 4–10 weeks, (2) 11–20 weeks, and (3) study period more than 20 weeks. Despite the broad study period in 33 studies, the positive effects of probiotic treatment on increasing beneficial bacteria and reducing cancer damage were demonstrated in all study periods, including the lowest and highest study periods. (4) cancer modeling agent with which optimal dosing is best? Since the optimal method and gold standard for tumor induction in CRC animal models have not yet been established, two major classes of cancer agents were discussed in these 33 studies, respectively: (1) AOM with a concentration of 8–12 mg/kg in a period between five days and six weeks, (2) 30 mg/kg and 40 mg/kg DMH used in rats in the induction period (2–10 weeks), as well as 10 mg/kg and 20 mg/kg in mice DMH (6–20 weeks). Since not all probiotics strains exhibit anti-CRC activities, screening the potent strain for the development of a probiotic-based therapeutic agent to control or prevent the incidence of CRC is crucial, and as regards, 85% of these 33 original studies used *Lactobacillus* species alone or mixed with other probiotics. With superior effects on CRC attenuation and successful modulation of the bacterial population in the microbiota, the *Lactobacillu*s genus can be suggested for comprehensive clinical studies in the treatment of colon cancer and promoting the reproduction of beneficial bacteria in the gut microbiota.

## 5. Conclusion

Since the precise mechanisms of probiotics for ameliorating human CRC as a multifactorial cancer, and circumstances of rapid shifts of the bacterial microbiota compositions from health to disease are still poorly understood, standard protocols must be applied in preclinical settings in order to ensure reproducibility and generalization of probiotics function results to clinical studies and develop treatment pathways based on a balance in fecal microbial structure. On the other hand, since animal metabolisms differ markedly from those of humans, animal models may not always reflect what occurs in humans. It is suggested to conduct additional studies, particularly long-term, randomized double-blind, placebo-controlled clinical trials to clarify and confirm preclinical findings in dealing with probiotics prior to advising their routine use as an adjunctive therapy for CRC prevention and treatment. Finally, the combination of CRC cell line studies, animal models, and clinical trials will help researchers develop a comprehensive picture of probiotic therapeutic pathways for guiding health care policies in the global fight against CRC.

## Figures and Tables

**Table 1:** Characteristics of the experimental animal models and probiotic intervention in studies of colorectal carcinogenesis.

First author (year)	Location	Animal	Number (age range)	Probiotics	Probiotic dose	Intervention	Cancer agent	Study period
Ali (2019)	Egypt	Swiss mice	40 (6 wks)	*L*. *casei* DSM 20011 (ATCC 393)	2 × 10^9^ CFU/0.3 ml per mice	Inulin	20 mg/kg DMH (20 wks)	24 wks
Arthur (2013)	USA	AOM/Il10^−/−^ mice	(7–12 wks)	VSL#3	10^9^ CFU/mice/day	No	10 mg/kg AOM (6 wks)	24 wks
Benguiar (2020)^*∗*^	Algeria	Wistar rats	ND	*B. lactis* LA 303, *L*. *acidophilus* LA 201, *L*. *plantarum* LA 301, *L*. *salivarius* LA (Lactic acid bacteria)	4 × 10^9^ CFU/g	Pomegranate peels	30 mg/kg DMH (9 wks)	16 wks
Brasiel (2021)	Brazil	Wistar rats	29	*Lactococcus and Lactobacillus* (kefir)	10^8^ CFU/mL	No	40 mg/kg DMH (2 wks)	24 wks
Chang (2018)	Taiwan	BALB/c mice	48 (6–8 wks)	*L*. *casei* (Lcr35)	1 × 10^7^ CFU/day	No	4 × 10^6^ CT26 cells	ND
Chen (2019)	China	Apc^min/+^ mice	90 (4 wks)	*C*. *butyricum* (ATCC 19398)	2 × 10^9^ CFU/0.2 mL	No	Apc^min/+^ mice	17 wks
Chou (2020)	Taiwan	ICR mice	30 (5 wks)	*L*. *fermentum* V3 *L. acidophilus* LA257 *L*. *rhamnosus* LR132	1 × 10^8^ CFU/day	No	10 mg/kg AOM (7 days) 2.5% DSS (7 days)	14 wks
Chung (2020)	South Korea	C57bL-6J mice	50 (8 wks)	*Pediococcus pentosaceus*	1 × 10^10^ CFU/ml	No	12.5 mg/kg AOM 2% DSS (5 days)	60 days
Cokasova (2012)	Slovak Republic	SD rats	33	*L*. *plantarum*	1 × 10^9^ CFU/ml	Oil (lini oleum virginale)	21 mg/kg DMH	8 months
Cruz (2020) (1)	Brazil	C57BL6/J mice	45 (8 wks)	VSL#3	2.25 × 10^9^ CFU/0.1 mL	Yacon	20 mg/kg DMH (8 wks)	13 wks
Cruz (2020) (2)	Brazil	IL-10^−/−^ mice	ND	VSL#3	10^9^ CFU/day	PBY (FOS and inulin)	20 mg/kg DMH (8 wks)	13 wks
Duarte (2020)	Italy	BALB/c mice	24 (12 wks)	*L*. *paracasei* DTA81 *L*. *rhamnosus* GG	3 × 10^9^ CFU/0.1 mL	No	20 mg/kg DMH	8 wks
Gamallat (2019)	China	SD rats	48 (4 wks)	*L*. *rhamnosus* LGG	1 × 10^9^ CFU/ml	No	40 mg/kg DMH (10 wks)	10 wks
Hakansson (2012)^*∗*^	Sweden	SD rats	48	*B*. *infantis* *L*. *gasseri* *L*. *plantarum*	2 × 10^9^ CFU/d 1 × 10^9^ CFU/d 3 × 10^9^ CFU/d	Blueberry husks	4% DSS (7 days)	6 months
Jacouton (2017)	France	C57BL/6 mice	(6–8 wks)	*L*. *casei* BL23	5 × 10^9^ CFU/ml	No	8 mg/kg AOM 2.5% DSS	46 days
Kuugbee (2016)^*∗*^	China	SD rats	40 (3 wks)	*L*. *acidophilus* *B*. *bifidum* and *B*. *infantum*	*L*. *acidophilus* (6.4 × 10^11^ cfu) *B*. *bifidum* & *B*. *infantum* (1.9 × 10^10^ cfu)	Fructo-oligosaccharide and maltodextrin	40 mg/kg DMH (10 wks)	26 wks
Liu (2020)	China	C57BL/6 mice	30 (8 wks)	*C*. *butyricum*	2 × 10^8^ CFU/ml	No	12.5 mg/kg AOM 2.5% DSS (14 days)	30 days
Mendes (2018)^*∗*^	Brazil	C57BL/6 mice	30 (8 wks)	Mixture (*L*. *acidophilus*, *L*. *rhamnosus*, *B*. *bifidum*)	0.6 × 10^9^ CFU/ml —	No	10 mg/kg AOM 2.5% DSS (5 days)	60 days
Ni (2016)	China	Apc^Min/+^ mice	ND	*L*. *rhamnosus* (LGG)	1 × 10^8^ CFU/day	No	Apc^min/+^ mice	16 wks
Peng (2020)	USA	Balb/c mice	60 (3 wks)	*L*. *casei*	1 × 10^9^ CFU/ml	Conjugated linoleic acids (CLA)	ND	28 days
Rong (2018)	China	C57BL/6 mice	120 (4-5 wks)	*L*. *helveticus* NS8	5 × 10^8^ CFU/ml	No	10 mg/kg AOM 3% DSS (7 days)	80 days
Saito (2019)^*∗*^	Japan	CPC; Apc mice	47 (7-8 wks)	*L. casei* strain shirota, *B*. *breve* strain yakult	1 × 10^8^ CFU/ml	*β*-Galactosyl-sucrose (prebiotic)	1% DSS (7 days)	20 wks
Song (2017)^*∗*^	China	C57BL/6 mice	35 (4 wks)	Bifico capsules containing *B*. *longum* L. *acidophilus* E. *faecalis*	1.2 × 10^7^ CFU/d (per mouse)	No	10 mg/kg AOM 2% DSS (7 days)	9 wks
Wang (2020)	China	C57BL/6 mice	31 (8 wks)	*B*. *bifidum* CGMCC 15068	3 × 10^9^ CFU/ml	No	10 mg/kg AOM 2% DSS (7 days)	10 wks
Wang (2021)^*∗*^	China	C57BL/6 mice	40 (6 wks)	*Lactiplantibacillus plantarum* KX041 *Lacticaseibacillus rhamnosus* LS8 *Loigolactobacillus coryniformis* MXJ32 *Companilactobacillus crustorum* MN047	1 × 10^9^ CFU/mL	No	10 mg/kg AOM (7 days) 2% DSS (7 days)	18 wks
Wang (2021)	China	C57BL/6 mice	40 (6 wks)	*L*. *coryniformis* MXJ32	1 × 10^9^ CFU/mL	No	10 mg/kg AOM (7 days) 2% DSS (7 days)	18 wks
Xu (2021)	Japan	C57BL/6NCrSlc mice	8 wks	*L. rhamnosus* probio-M9	2 × 10^9^ CFU/day	No	12 mg/kg AOM 2% DSS (7 days)	20 wks
Yuan (2018)^*∗*^	China	BALB/c mice	32 (6–8 wks)	*Bifidobacterium* and *Lactobacillus tablets*	ND	ABX	2 × 10^6^ CT26 cells	33 days
Yue (2020)	China	APC^Min/+^ mice	36 (6 wks)	*L*. *plantarum* YYC-3 Cell-free supernatant of *L*. *plantarum* YYC-3 (YYCS)	1 × 10^9^ CFU/ml	No	APC^Min/+^mice	7 wks
Zhang (2015)	China	F344 rats	24 (5 wks)	*L*. *salivarius* Ren	5 × 10^10^ CFU/kg b.w/d	No	30 mg/kg DMH (10 wks)	32 wks
Zhang (2017)	China	C57BL/6J mice	24	*L*. *casei* Zhang	4 × 10^9^ CFU/d	Vitamin K2 (menaquinone- 7)	12.5 mg/kg AOM (7 days) 2.5% DSS (5 days)	ND
Zhu (2014)	China	F344 rats	50 (5 wks)	*L*. *salivarius Ren*	High dose (2 × 10^9^ CFU/rat) Low dose (1 × 10^8^ CFU/rat)	No	30 mg/kg DMH (10 wks)	15 wks
Zhuo (2019)	China	BALB/c mice	50 (6–8 wks)	Lysates of *L*. *acidophilus*	High dose (2 × 10^7^ CFU/mouse)	CTLA-4 IgG (50 *μ*g/mouse)	10 mg/kg AOM (7 days) 2% DSS (5 days)	12 wks

^*∗*^ shows studies that used a combination of multistrain probiotic bacteria. *L*, *Lactobacillus; B*, *Bifidobacterium*; ND, not determined; wks, weeks.

**Table 2:** Gut microbiota, SCFAs, and fecal enzyme changes by probiotic intervention in CRC-animal models.

Study	Pro and/or + prebiotic + cancer	SCFAs	Fecal enzymes	Signaling pathway		Microbiota composition	
				Increase	Decrease	Increase	Decrease
^1^Peng et al. [47]	*L. casei* *+* conjugated linoleic acids (CLA)					In phylum *Firmicutes, Thermotogae* in LC-WT and LC-CLA At genus *Bifidobacterium, Lactobacillus* in LC-WT and LC-CLA	In phylum *Bacteroidetes, Proteobacteria, Verrucomicrobia* in LC-WT and LC-CLA At genus *Bacteroides* in LC-WT and LC-CLA

^1^Liu et al. [23]	*C. butyricum* + AOM/DSS			Bax	p-I*κ*B*α* NF-*κ*B Bcl-2	At phylum *Bacteroidetes* At genus *Prevotella, Lactobacillus, Turicibacter, Allobaculum, Sutterella, Butyricimonas, Barnesiella*	At phylum *Firmicutes* At family Lachospiraceae
							At genus *Oscillospira, Anaeroplasma*

^1^Yuan et al. [30]	*Bifidobacterium and* *Lactobacillus* tablets + FU + CT-26					At genus *Alloprevotella, Citrobacter, Prevotellaceae_UCG-001, Roseburia, Thalassospira, Erysipelatoclostridium,* Lachnospiraceae*_UCG-006* In species *Bacteroides_chinchillae, Helicobacter ganmani*	At genus *Desulfovibrio, Anaerotruncus, Mucispirillum Odoribacter, Ecoli-Shigella* In species *Bacteroides vulgatus,* Lachnospiraceae*_bacterium_1* 0-1

^1^Yue et al. [31]	*L. plantarum* YYC-3 (live), cell-free supernatant (YYCS) + C57BL/6-APC^Min/+^				*β*-Catenin I*κ*B*α* P-65	In phylum *Firmicutes*, *Actinobacteria* (YYC-3)	

^1^Jacouton et al. [45]	*L. casei* BL23 + AOM/DSS			Caspase-7 Caspase-9 Bik		At phylum *Firmicutes*	
						At family Ruminococcaceae	
						At genus *Prevotella, Lactobacillus*	
						In species *Lactobacillus zeae*	

^1^Chung et al. [40]	*P. pentosaceus* + AOM/DSS					At family Akkermansiaceae, Lachnospiraceae, Lactobacillaceae, Ruminococcaceae, Oscillibacter	At family *Erysipelotrichaceae, Turicibacter*

^1^da Silva Duarte et al. [42]	*L. paracasei DTA81* *+* DMH *L. rhamnosus GG* *+* DMH	(H^#^) acetic acid & propionic acid (H^#^) butyric acid (H^#^) total SCFAs		P53 caspase 3 (NS)		In genus *Ruminiclostridium* in DTA81 *Romboutsia* and *Turicibacter* in LGG	

^1^Mendes et al. [46]	(*L. acidophilus* *+* *L.rhamnosus* *+* *B. bifidum*) + AOM/DSS					At phylum *Actinobacteria*	At genus *Clostridium XIVa*
						At genus *Lactobacillus, Bifidobacterium, Akkermansia, Allobaculum, Clostridium XI, Clostridium XVII*	

^1^Song et al. [26]	*Bifico* (*B. longum L. acidophilus E. faecalis*) + AOM/DSS					At genus *Lactobacillus*	In phylum *Deferribacteres*
							At genus *Mucispirillum, Desulfovibrio, Odoribacter*

^1^Wang et al. [27]	*B. bifidum* + AOM/DSS					In phylum *Proteobacteria, Verrucomicrobia*	In phylum Bacteroidetes, Firmicutes, Actinobacteria …
						At family Desulfovibrionaceae, Verrucomicrobiaceae, Ruminococcaceae, Lachnospiraceae	
						At genus *Romboutsia, Turicibacter, Lactobacillus, Akkermansia*	

^2^Rong et al. [25]	*L. helveticus NS8* + AOM/DSS				*β*-Catenin Cox-2 p-I*κ*B*α*	At family Bacteroidaceae In species *Bacteroides acidifaciens, Lactobacillus* spp. *Bifidobacterium pseudolongum*	In phylum Cyanobacteria, Candidatus, Saccharibacteria Deferribacteres
							At family Porphyromonadaceae, Prevotellaceae
							At genus *Prevotella*
							In species *Bacteroides uniformis, Odoribacter* spp.

^2^Zhuo et al. [35]	*L.acidophilus* *+* CTLA-4 mAb + AOM/DSS						In phylum *Proteobacteria*

^2^Cruz et al. [50] (1)	*VSL#3* + Yacon + DMH	(H^#^) Acetic acid (H^#^) Propionic acid (H^#^) Butyric acid (in SYN) (L^†^) *β*-glucuronidase (in SYN)				In phylum *Firmicutes* in PRO	In phylum Firmicutes in SYN Bacteroidetes, Proteobacteria in PRO and SYN
						At family Lactobacillaceae, Turicibacteraceae in PRO Lachnospiraceae in SYN Clostridiaceae in PRO & SYN	At family *Helicobacteraceae* in PRO & SYN
						In genus *Lactobacillus, Allobaculum, Streptococcus* in PRO and SYN *Bifidobacterium, Roseburia, Blautia, Gemella, 02d06* in PRO *Clostridium* in SYN	In genus *Coprococcus, Dorea, Flexispira, Oscillospira* in PRO *Ruminococcus, Butyrivibrio, Sutterella, Helicobacter* in PRO, *Brachyspira* in SYN

^2^Cruz et al. [50] (2)	*VSL#3* + PBY + DMH	(H^#^) Acetic acid, Propionic acid (H^#^) Butyric acid (in SYN)				In phylum *Proteobacteria, Actinobacteria*	In phylum *Patescibacteria, Firmicutes, Bacteroidetes*
						At family Desulfovibrionaceae, Legionellaceae Erysipelotrichaceae	At family Saccharimonadaceae, Ruminococcaceae Lachnospiraceae

^2^Chou et al. [39]	*L. fermentum* *+* AOM/DSS					At order *Lactobacillales*	At phylum *Bacteroidetes* At genus *Akkermansia*
						In species *Lactobacillus fermentum*	
						At genus *Lactobacillus*	

^2^Zhu et al. [34] 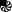	*L. salivarius Ren* + DMH	(H^#^) acetic (H^#^) propionic (H^#^) butyric				In species *Clostridium bifermentans Lachnospiraceae bacterium, Prevotella* spp.	In species *Bacillus subtilis*, *Uncultured Ruminococcaceae bacterium*
			(L^#^) Azoreductase (NS^2^) *β*-glucosidase (NS^2^) *β*-glucuronidase				

^2^Benguiar et al. [37]	*B. lactis, L. acidophilus, L. plantarum, L. salivarius, B. lactis* + pomegranate peels + DMH					In species *Lactobacillus* spp.*, Bifidobacterium* spp.	In species *E.coli, Bacteroides* spp.

^2^Ni et al. [24]	*L. rhamnosus (LGG)* *+* Apc^min/+^	(H^#^) Butyric acid (H^#^) Propionic acid				At genus *Coprococcus, Alistipes, Anaerostipes Roseburia, Blautia*	At genus *Akkermansia*

^2^Chen et al. [20]	*C. butyricum* + Apc^min/+^	(H^#^) Acetic acid (H^#^) Propionic acid (H^#^)Butyric acid			*β*-Catenin	At family Ruminococcaceae	At genus *Desulfovibrio, Odoribacter, Helicobacter*
						At genus *Eubacterium*	

^2^Wang et al. [28] (1)	(*L. plantarum* *+* *L. rhamnosus* *+* *L. coryniformis* *+* *C. crustorum*) + AOM/DSS	(H^#^) Butyric acid				In phylum *Proteobacteria*	In genus *Turicibacter, Ruminococcaceae_UCG-014, Candidatus_Saccharimonas, Desulfovibrio, Bacteroides, uncultured_bacterium_o_Mollicutes_RF39, Parabacteroides*
						In genus *Lachnospiraceae_NK4A136_group, Faecalibaculum, Roseburia, Blautia, Lachnoclostridium, uncultured_bacterium_f_ Lachnospiraceae*	

^2^Wang et al. [28] (2)	*L. coryniformis* MXJ32 + AOM/DSS	(H^#^) Total SCFAs				In genus *Lactobacillus, Bifdobacterium, Akkermansia, Faecalibaculum*	In genus *Desulfovibrio, Helicobacter*

^2^Saito et al. [52]	(*L. casei strain Shirota* *+* *B. breve strain Yakult*) *+* *β*-galactosyl- sucrose + DSS					In species *L. casei, B. breve* in *PRO & SYN*	

^2^Xu et al. [48]	*L. rhamnosus* Probio-M9 *+* AOM/DSS					In genus *Blautia, Akkermansia, Bifidobacterium*	

^3^Ali et al. [49]	*L. casei* + Inulin + DMH			p-JNK-1	*β*-catenin p-GSK3*β*	At phylum *Verrucomicrobia* in PRO & SYN	
						At genus *Akkermansia* in PRO & SYN *Turicibacter* in PRO & SYN	

^3^Arthur et al. [36]	*VSL#3* + AOM					At phylum *Proteobacteria* (mucus microbiota)	At phylum *Verrucomicrobia* (mucus microbiota) *Bacteroidetes* (stool microbiota)
							In family *Porphyromonadaceae, Verrucomicrobiaceae* (mucus & stool)

^3^de Almeida Brasiel et al. [43]	Kefir + DMH					At genus *Lactobacillus* in KSL *Romboutsia* in KNL and KSL	At genus *Prevotellaceae_NK3B31 in* KSL & KNL *Acinetobacter in* KSL

^3^Chang et al. [38]	*L. casei* (*Lcr35*) + FOLFOX + CT-26			Bax/Bcl-2 Caspase-8 (NS)		At phylum *Bacteroidetes in FOLFOX* *+* *Lcr35* in compared to FOLFOX	At phylum *Firmicutes* in FOLFOX + *Lcr35* in compared to FOLFOX

^3^Čokášová et al. [41]	*L. plantarum* + oil + DMH		(L^†^) *β*-glucuronidase (L^†^) *β*-galactosidase			*lactobacilli*	*coliforms*

^3^Gamallat et al. [21]	*L. rhamnosus* (*LGG*) + DMH			Bax Caspase-3 P53	Bcl-2	At family Lactobacillaceae, Corynebacteriaceae, Provetellaceae, Bacteriodaceae, Clostridiaceae At genus *Lactobacillus*	

^3^Kuugbee et al. [22]	(*L. acidophilus* *+* *B. bifidum B. infantum*) + FOS *+* DMH				Caspase-3 Cox-2 *β*-catenin	At genus *Lactobacillus*	At phylum *Proteobacteria*, *Chlamydiae*, *Bacteroidetes*
							At genus *Escherichia, Helicobacter, Clostridium, Pseudomonas*

^3^Zhang et al. [32] 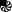	*L. salivarius Ren* + DMH					In species *Prevotella* spp.	In species *Bacteroides dorei*, *Ruminococcus sp. Clostridiales bacterium*

^3^Zhang et al. [33]	*L. casei.*Zhang + VK + AOM/DSS	(H^#^) Acetic acid (H^#^) Butyric acid		Caspase-3		In phylum *Deferribacteres, Bacteroidetes*	In phylum *Verrucomicrobia*
						At family Prevotellaceae	
						In species *Alloprevotella rava, Parabacteroides merdae*	

Microbiome bacterial population, SCFAs levels, and bacterial enzyme activities were changed by probiotic supplementation in CRC animal groups. ^
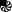
^ shows that studies used denaturing gradient gel electrophoresis (DGGE) method to microbiota composition analysis; H^#^, high level; L^†^, low level; NS^2^, not significant. Microbiome composition, SCFAs, and bacterial enzymes were compared between cancer groups and pro/synbiotic treated cancer groups in the included studies. ^1^ shows study period between 4 and 10 weeks; ^2^ shows study period between 11 and 20 weeks; ^3^ shows study period more than 20 weeks.
